# Sensory Properties and Aroma Compound Profiles of Pea Protein Isolates in Relation to High Hydrostatic Pressure and High‐Pressure Homogenization Treatments

**DOI:** 10.1002/fsn3.71889

**Published:** 2026-05-27

**Authors:** Lea Friedrich, Christina Hopf, Laura Scheuer, Pauline Fauquet, Gina Zeh

**Affiliations:** ^1^ Fraunhofer Institute for Process Engineering and Packaging IVV Freising Germany; ^2^ Department of Chemistry and Pharmacy, Chair of Aroma and Smell Research, Friedrich‐Alexander‐Universität Erlangen‐Nürnberg Erlangen Germany; ^3^ TUM School of Life Sciences Weihenstephan, Technical University of Munich Freising Germany; ^4^ Food Department Celabor Herve Belgium

**Keywords:** aroma analysis, flavor profile, Gas Chromatography–Mass Spectrometry (GC–MS), Gas Chromatography‐Olfactometry (GC–O), sensory analysis

## Abstract

Plant‐derived proteins are becoming increasingly important due to consumer demand for plant‐based alternatives to animal products. Processing of these proteins can negatively affect their flavor. While there have been many investigations into the role of thermal processing, the impact of high‐pressure treatment (i.e., high hydrostatic pressure and high‐pressure homogenization) on the flavor profile of plant‐derived proteins is still unclear. In the present work, the sensory properties and aroma compound profiles of commercial pea protein isolates were systematically investigated via descriptive sensory analysis and instrumental aroma analysis in relation to high‐pressure treatments (before and after treatment). The flavor profiles of the protein isolates were described as mainly *pea‐like/bean‐like, flour‐like/cereal‐like, bitter, fatty*, and *sour*, with no notable differences before and after high‐pressure treatments. Gas chromatography‐olfactometry/mass spectrometry revealed the presence of unsaturated aldehydes, typical products of lipid oxidation, associated with the overall *fatty, green* aroma, but the compositions did not change significantly through high‐pressure treatment, suggesting the suitability of HPH or HHP for processing or preservation of pea protein isolates without affecting the flavor profile.

## Graphical abstract

Flavor profiles of untreated pea protein isolate (light green, solid line) compared to High Pressure Homogenization (HPH) treated (dark blue, dash‐dot line) and High Hydrostatic Pressure (HHP) treated pea protein isolates (dark green, dashed line). Samples were described as mainly pea‐like/bean‐like, flour‐like/cereal‐like, bitter, fatty, and sour. Unsaturated aldehydes hexanal (grassy), (*E,Z*)‐2,4‐nonadienal (fatty), and (*E,E*)‐2,4‐heptadienal (fatty, fruity), as well as the compounds 3‐isobutyl‐2‐methoxypyrazine (bell pepper‐like, pea‐like) and sotolone (savory, celery‐like) were identified as major contributors to the overall aroma. Neither of the pressure treatments significantly changed the overall flavor profile of the pea protein isolate.

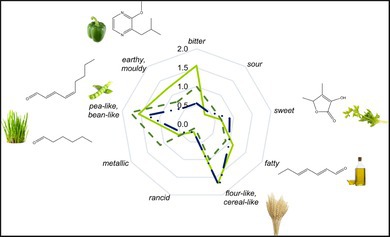

## 1 Introduction

There is a growing demand for plant‐based alternatives to animal products (i.e., meat, fish, and dairy) due to a rising consumer awareness for maintaining a healthy diet and choosing more sustainable foods (García Arteaga et al. 2020; Gläser et al. 2020; Jeske et al. 2019, 2017; Lam et al. 2018; Sethi et al. 2016; Shanthakumar et al. 2022). The production of plant‐based alternatives requires protein‐rich raw materials, including legumes, such as soy or pea (Wang et al. 2022). The use of pea ( *Pisum sativum* ) as a protein source has several advantages. Pea is a legume that grows in temperate climates, thus it can be produced in central Europe (Neugschwandtner et al. 2020) without the need for long transportation routes. Cultivation of pea pulses is inexpensive and has a low water consumption (Sim et al. 2019). Moreover, peas have a low allergenic potential (Barac et al. 2010; Ge et al. 2020; Lam et al. 2018; Stone et al. 2015) and are highly nutritious, with a protein content of 18%–32% (Boye et al. 2010; Roy et al. 2010).

Nevertheless, the use of pea protein in developing plant‐based alternative products presents a challenge due to its characteristic flavor (Lan et al. 2019), which is often perceived as an off‐flavor by consumers and can reduce the acceptance of foods derived from this protein (Roland et al. 2017). Several factors come into play with regard to the undesirable flavor of pea protein, including the inherent aroma‐active compounds that are present in the pea at the time of harvest, and compounds that are formed after harvest, for example, during protein extraction and isolation or its further processing, or through storage (Liu et al. 2023; Roland et al. 2017; Sessa and Rackis 1977).

The inherent aroma‐active compounds include, in particular, the pyrazines, which exhibit *green, green bell‐pepper‐like, pea‐like, vegetable‐like* aroma (Heng 2005; Murat et al. 2013; Roland et al. 2017; Trikusuma et al. 2020). During storage (Mehle et al. 2020) or processing, *grassy, beany* aldehydes from lipid oxidation particularly impact the aroma of pea protein (Trikusuma et al. 2020). Furthermore, products of the Strecker degradation and the Maillard reaction (methional, 2‐methylpropanal, 2‐methylbutanal, phenylacetaldehyde (Liu et al. 2023)), 2‐acetylpyrroline contribute to the aroma profile (Liu et al. 2023; Trikusuma et al. 2020). In addition, pyrazines can also be formed by the Maillard reaction upon heat treatment (Liu et al. 2023; Ma et al. 2016; Trikusuma et al. 2020).

Moreover, the aroma profile and aroma constituents can be affected by processing (i.e., extraction, preservation, drying), which typically involves heating procedures, and subsequent storage in different ways (Fischer et al. 2022; Heng 2005; Liu et al. 2023; Mehle et al. 2020; Murat et al. 2013). While some aroma compounds in pea flour or pea protein can be removed or reduced by heat treatment, other compounds can be formed at elevated temperatures, especially Strecker, Maillard or decarboxylation products (Ebert et al. 2022; Ma et al. 2016; Murat et al. 2013). Consequently, heat treatment does not necessarily lead to a reduction but also not always an enhancement of off‐flavors. Depending on the composition of the peas and/or pea proteins, temperature and exposure time, thermal treatment can either help to reduce off‐flavors or can lead to the formation of undesirable aroma compounds (Azarnia et al. 2011; Ebert et al. 2022; Ma et al. 2016; Trikusuma et al. 2020; Trindler et al. 2022; Vatansever et al. 2024). In addition, heat treatment can deteriorate the protein functionality (Vatansever et al. 2024). Besides aroma compound formation and degradation, the interactions between aroma compounds and the food matrix play a major role in the overall aroma profile of foods. The interactions between proteins and flavor compounds have been investigated in many studies, but they are complex and some aspects remain unclear. Non‐covalent interactions like hydrophobic interactions occur, for example, between the nonpolar side chains of aldehydes or ketones and hydrophobic pockets in the protein structure. More polar molecules or functional groups can form hydrogen bonds with more hydrophilic protein areas (Barallat‐Pérez et al. 2023; Dai et al. 2026). Covalent bonds can be formed by reaction of amino groups of the side chains of lysine or arginine with carbonyl groups of aldehydes, or by reaction of free sulfhydryl groups with disulfides (Wang and Arntfield 2017).

The interactions between protein and aroma‐active substances can have both positive and negative effects on the flavor properties of the protein: Permanent retention of undesirable aroma substances can reduce the perceived off‐flavors of a protein and improve the overall flavor profile (Vatansever et al. 2024). If, on the other hand, undesirable flavor compounds are only temporarily retained during processing, but no longer during storage or consumption, they cannot be removed in the process but can still impair the perception of the food. Another negative effect would be the retention of potentially added, desirable flavorings, such as vanillin (Dai et al. 2026).

While there are numerous studies on the influence of temperature on the sensory properties of pea protein, knowledge regarding the influence of high‐pressure processes is hitherto still incomplete. High‐pressure processes are widely used in the food industry, for example, high‐pressure homogenization (HPH) (up to 350 MPa) (Bader et al. 2011; Melchior et al. 2022) or high‐pressure pasteurization/high hydrostatic pressure (HHP) (San Martín et al. 2002; Shanthakumar et al. 2022). Usually, high‐pressure processes, such as HPH and HHP, cannot break covalent bonds, but they can alter the weaker interactions between aroma‐active compounds and proteins (Vatansever et al. 2024). A very important application of high‐pressure treatment is the modification of the techno‐functional properties of plant‐based proteins (Scheuer et al. 2026). HPH in the range of approximately 100 MPa led to improved solubility in various proteins. For example, faba beans, lentils, and chickpeas (Ma et al. 2023; Saricaoglu 2020; Yang et al. 2018). In lupine, chickpea, and lentil protein, HPH treatment in the range of approximately 100 MPa also improved the emulsifying properties (Bader et al. 2011; Ma et al. 2023; Saricaoglu 2020), but in faba bean protein and lentil protein at over 100 MPa, a deterioration in emulsifying properties was observed (Saricaoglu 2020; Yang et al. 2018). HPH treatment also led to an increase in gel strength, emulsion stability, and foaming capacity in some cases (Bader et al. 2011; Ma et al. 2023; Saricaoglu 2020; Yang et al. 2018). Similarly, in pea protein, HPH led to an increase in solubility, foaming and emulsifying capacity, water and oil holding capacity, and emulsion stability. In various studies, this improvement in properties occurred at 30, 50, or 70 MPa; at pressures above 70 MPa, aggregation of the protein and deterioration of properties was observed in some cases, but in others, properties improved even at 100 or 200 Mpa (D'Alessio et al. 2023; Levy et al. 2025; Luo et al. 2022; Melchior et al. 2022; Scheuer et al. 2026). In terms of technological properties, according to the literature, pressure treatment of plant proteins such as pea protein would therefore be highly beneficial. However, it is not yet clear whether and how this would alter the sensory properties. Thermal treatment of pea protein can change its sensory properties, in a positive or negative way. In contrast to thermal treatments, high pressure is considered beneficial regarding the protein functionality and gentle in terms of sensory properties, but this has not yet been properly investigated for pea protein (Vatansever et al. 2024). Instead, most studies on pressure treatment of plant proteins to date have focused on techno‐functional properties or solely targeted the sensory analysis of soy protein. Contrary to the growing use of pea protein in food products (Trindler et al. 2022), the effects of high pressure on the flavor of pea protein remains underexplored (Lomelí‐Martín et al. 2021; Vatansever et al. 2024).

In the present work, a combined sensory and analytical approach was employed to compare the sensory properties and the underlying flavor compounds of different pea protein isolates, in relation to high‐pressure treatments. The aim of this work was to investigate the degree to which high‐pressure processing, specifically HPH or HHP, on pea protein isolates affects their sensory profiles and aroma compound constituents. Insights from this work will serve as a basis for further research on how processing technologies affect the flavor of pea proteins and provide indications on how off‐flavors may be avoided.

## 2 Materials and Methods

### 2.1 Chemicals

Dichloromethane (DCM), technical grade (≥ 98%; VWR, Darmstadt, Germany) was distilled for purification prior to use. Anhydrous sodium sulfate p.a. (Merck, Darmstadt, Germany) was dried overnight at 105°C before use. For the instrumental analyses, helium (> 99.996%, Linde GmbH, Pullach, Germany) was used as carrier gas. Samples for sensory evaluation and analytical evaluation were prepared using tap water.

### 2.2 Sample Material

Four commercial pea protein isolates (PPI) (P1, P2, P3, P4) were obtained from two different European manufacturers. P1 is sold as a functional standard PPI for various food applications. P2 is sold as a high‐viscous PPI for texturization. P3 is sold for use in meat alternatives, spreads, and sauces. P4 is sold for use in bakery products and cereals (cf. Table 1). All four PPI are produced from yellow pea ( *Pisum sativum* ). There is no information available regarding the pea cultivar, as it is not specified by the manufacturers. According to the manufacturer, P3 and P4 are obtained from peas sourced in Europe in an aqueous extraction process using acids and bases, without the use of organic solvents. For P1 and P2, there is no information available about the origin of crops or the extraction process. The two pressure‐treated samples P1‐HPH and P1‐HHP have been produced from P1 at the research facilities Fraunhofer IVV and Celabor, respectively. The exact processing parameters can be found in Table 1.

**TABLE 1:** Investigated PPI samples.

Sample code	Parameters	Origin	Use case
P1		Commercial	Various food applications
P1‐HPH	P1 homogenized at high pressure (60 MPa, 3 cycles)	Fraunhofer IVV, Germany, from commercial product P1	
P1‐HHP	P1 processed with high hydrostatic pressure (600 MPa, 10 min)	Celabor, Belgium, from commercial product P1	
P2		Commercial	Texturization
P3		Commercial	Meat alternatives, spreads, sauces
P4		Commercial	Bakery products, cereals

### 2.3 Sensory Evaluation

The four commercial PPI (P1, P2, P3, P4) were evaluated by descriptive sensory analysis based on consensus profiling according to DIN 10967‐2:2000‐10 (Deutsches Institut für Normung 2000). The analysis was executed at Fraunhofer IVV with ten trained panelists (7 female, 3 male, aged 23–56 years) in single determination. Training was performed according to DIN 10961:1996‐08 (Deutsches Institut für Normung 1996).

The ideal concentration of the PPI samples in water for sensory evaluation was determined in preliminary tests. A concentration of 0.6% (w/w) PPI in water optimally balanced the perception of the overall aroma and, at the same time, allowed the detection of aroma differences in subtle nuances. Therefore, this concentration was chosen for the following sensory analyses. The pH value of 0.6% (w/w) PPI P1—P4 in water was determined to be 7.06, 7.50, 7.31, and 5.54, respectively.

Aliquots of about 20 g of 0.6% (w/w) pea protein solution were served in a randomized order in glasses with lids labeled with random three‐digit numbers. Water and toast were served and the panelists were instructed to use both to cleanse their palate to neutralize any remaining sensory perception in the time gaps (30 s) between samples. Retasting of samples was permitted.

The panelists were asked to evaluate the products in terms of taste and retronasal aroma. Evaluation took place in a neutral room. They evaluated all samples individually first (communication between participants was prohibited in this part of the evaluation), then the attributes were collected. Similar attributes, like flour‐like and cereal‐like, were combined. Hedonic terms and attributes that applied equally to all samples were discarded. Attributes present in every sample but differing in intensity were maintained. Next, the intensities for the decided attributes were assessed individually (communication between participants was prohibited in this part of the evaluation) on a scale from zero to three (not detectable, weakly detectable, clearly detectable, strongly detectable). All results are expressed as mean values. One‐way analysis of variance (ANOVA; *α* = 0.05) was performed on the sensory data to check for descriptors that significantly differentiated between the samples. The mean values were compared using Duncan's post hoc test. XLSTAT software (Lumivero, Denver, USA) was used for statistical evaluation of the sensory data. The homogeneity of variance/homoscedasticity was tested using the Levene test: If any of the residuals were heteroscedastic, a Brown‐Forsythe test was additionally performed. The normal distribution of the residuals was evaluated using the Shapiro–Wilk test: If any of the residuals did not follow a normal distribution, a non‐parametric Kruskal‐Wallis test was performed in addition.

Two pressure‐treated PPIs and the corresponding untreated PPI were evaluated by descriptive sensory analysis in another session following the same procedure, with eight trained panelists (6 female, 2 male, aged 23–56 years). Each panelist was placed individually in a neutral room (temperature 22°C ± 1°C). The temperature of the samples was also 22°C ± 1°C.

### 2.4 Aroma Analysis

#### 2.4.1 Isolation of Selected Aroma Compounds

The four commercial PPIs were obtained as a dry powder. To 50.00 g (±0.05 g) of each dry pea protein isolate, 50 mL of water was added in an Erlenmeyer flask (500 mL). After closing the flasks with a stopper, the protein isolate was let to soak at room temperature for 10 min. The pH value of soaked PPI P1—P4 was determined to be 6.95, 7.74, 7.14, and 5.43, respectively. After addition of 150 mL of DCM and constant stirring at RT for 45 min, the solution was filtered through a folded qualitative filter paper (240 mm, VWR International, Leuven, Belgium). The filter cake was rinsed with DCM (2 × 75 mL). The combined organic layers were dried over anhydrous sodium sulfate, filtered, and subjected to high vacuum distillation via solvent‐assisted flavor evaporation (SAFE) technique to separate volatiles from non‐volatiles (Engel et al. 1999). The distillate was concentrated to ~5 mL at 47°C on a rotary evaporator (IKA, Staufen, Germany) and finally to a volume of ~100 μL using micro distillation at 47°C (Bemelmans 1979). A blank sample was obtained by extracting 50 mL of deionized water with 150 mL DCM in accordance with the extraction of raw PPI (see above). For SAFE distillation, the blank samples were run through the SAFE apparatus without performing the distillation.

HPH‐processed PPI was obtained as a 10% solution. Therefore, 500 g of the 10% P1‐HPH solution was used as sample material. It was extracted in two batches. Each batch was extracted as described for the commercial PPIs, but the matrix was not separated by filtering.

HHP‐processed PPI was obtained as a 15% solution. Therefore, 333 g of the 15% P1‐HHP solution was used as sample material. It was weighed equally into 10 40 mL centrifugal tubes and centrifuged at 27000 × *g*, 15°C for 30 min. The aqueous supernatant was discarded, and the PPI residues were collected in an Erlenmeyer flask closed with a stopper (500 mL). Then, 300 mL of DCM were added for solvent extraction and stirred on a magnetic stirrer for 45 min, filtered through a paper filter, and the flask and residue were washed with 300 mL of DCM. The combined organic layers were dried over anhydrous sodium sulfate, filtered, and subjected to high vacuum distillation via the SAFE technique to separate volatiles from non‐volatiles (Engel et al. 1999). The distillate was concentrated to ~5 mL at 47°C on a rotary evaporator (IKA, Staufen, Germany) and finally to a volume of ~100 μL using micro distillation at 47°C (Bemelmans 1979). Sample extraction was performed in a single determination.

#### 2.4.2 Gas Chromatography‐Olfactometry (GC–O)

The obtained distillates (Section 2.4.1) were analyzed by means of gas chromatography‐olfactometry (GC–O). The analyzing system was assembled of a Trace GC Ultra (Thermo Fisher, Waltham MA, USA) using a deactivated (DPTMDS) precolumn (5 m × 0.53 mm; Chromatographie‐Zubehoer Trott, Kriftel, Germany) and a DB‐FFAP fused silica capillary column (30 m × 0.25 mm ID, 0.25 μm; J & W Scientific, USA). Helium was used as carrier gas with a constant flow rate of 2.2 mL min^−1^. Aliquots (2 μL) of the sample distillates were injected manually via the cold on‐column technique (40°C). The initial temperature was held at 40°C for 2 min before it was raised gradually at a rate of 8°C min^−1^ to 235°C, and then held at 235°C for 5 min. The effluent was split in a 1:1 volume ratio by a Y‐splitter and transferred to a flame ionization detector (FID; Thermo Fisher, Waltham MA, USA) and an odor detection port (ODP; self‐made) via two uncoated, deactivated fused silica capillaries (0.7 m × 0.2 mm). The FID signal was recorded by an analogue recorder. This allowed for simultaneous recording of a chromatogram and detection of the aroma‐active regions within the chromatogram. The temperature of the FID and the ODP were set to 250°C and 230°C, respectively. Linear retention indices (RIs) of the aroma compounds were calculated (Van den Dool and Kratz 1963). In addition, each of the distillates obtained was chromatographed and sniffed on a chromatography column with different polarity (DB‐5).

#### 2.4.3 Aroma Extract Dilution Analysis (AEDA)

The aroma distillate (cf. Section 2.4.1) was diluted stepwise (1 + 1, v + v) with DCM, resulting in solutions corresponding to flavor dilution (FD) factors up to 2048. The FD factors of the single odorants were determined using GC‐O, representing the last dilution in which the individual odorant was still perceivable (Grosch 1993). GC‐O was performed on the undiluted distillate (FD 1) by three trained panelists (cf. Section 2.3) to avoid overlooking odorants, for instance due to partial anosmia or lower sensitivities to particular substances. An aroma‐active region was considered if it was perceived by ≥ 2 of 3 panelists. Apart from the different panelists in FD 1, the AEDA was performed in a single determination.

#### 2.4.4 Gas Chromatography–Mass Spectrometry/Olfactometry (GC–MS/O)

The obtained distillates (cf. Section 2.4.1) were analyzed by means of GC–MS/O. The system was assembled of an Agilent 7890B gas chromatograph, coupled to an Agilent 5977B Series MSD mass spectrometer (Agilent, Waldbronn, Germany) and an ODP (Gerstel, Mühlheim, Germany), using a deactivated (DPTMDS) pre‐column (5 m × 0.53 mm; Chromatographie Zubehör Trott, Kriftel, Germany) and a DB‐FFAP fused silica capillary column (30 m × 0.25 mm ID, 0.25 μm; J & W Scientific, USA). Helium was used as carrier gas with a constant flow rate of 2.2 mL min^−1^. Aliquots (2 μL) of the sample distillates were injected automatically by a MPS autosampler (Gerstel, Mühlheim, Germany) into a Multimode Inlet (Agilent, Waldbronn, Germany) at 280°C. The initial temperature on the column was held at 40°C for 2 min before it was raised gradually at a rate of 8°C min^−1^ to 235°C, and then held for 5 min at 235°C. The effluent was split by a Y‐splitter and transferred to the MS and the ODP via two uncoated, deactivated fused silica capillaries (0.7 m × 0.2 mm). The transfer line temperature to the MS was set to 250°C. Mass spectra in the electron ionization (EI) mode were generated at 70 eV in full scan mode (*m*/*z* 35–249). Linear retention indices (RIs) of the aroma compounds were calculated (Van den Dool and Kratz 1963). Identification of aroma‐active compounds involved comparing odor impressions, RIs on both columns, and mass spectra, using the Fraunhofer IVV internal reference substance library. The resulting library matches were additionally cross‐checked using the NIST database (version NIST17, NIST Mass Spectrometry Data Center). If for an aroma‐active region a substance could not be identified by at least the RI on one column and the odor impression, this substance was considered as “unknown”.

## 3 Results

### 3.1 Sensory Evaluation

**FIGURE 1:**
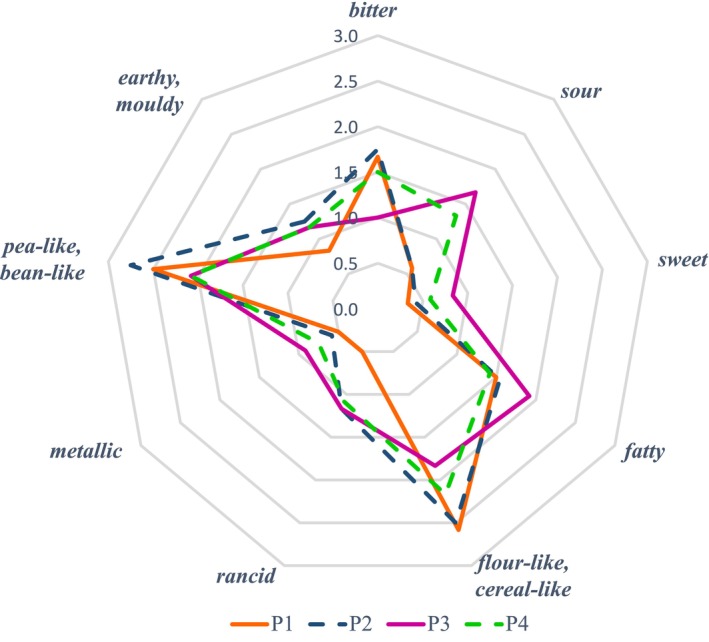
Comparison of intensity of aroma attributes for four different non‐pressure‐treated pea protein isolates (PPI).

Consensus profiling of the four non‐pressure‐treated PPI revealed the taste attributes *bitter*, *sour* and *sweet* and the retronasal aroma attributes *fatty*, *flour‐like/cereal‐like*, *rancid*, *metallic*, *pea‐like/bean‐like* and *earthy/moldy*. Attributes perceived with highest intensities are *pea‐like/bean‐like*, *flour‐like/cereal‐like*, *bitter*, *fatty* and *sour* (cf. Figure 1). Figure 1 further shows that in total, the four PPI have a similar flavor profile. Statistical ANOVA (*α* = 0.05) showed no significant differences between the samples for all attributes except for *sour* (*p* = 0.037). The normal distribution of the residuals was examined using the Shapiro–Wilk test: For all 9 variables, the residuals do not follow a normal distribution. Therefore, a non‐parametric Kruskal‐Wallis test (*α* = 0.05) was performed in addition, which found no significant differences between the samples for all attributes. The homogeneity of variance/homoscedasticity was examined using the Levene test: The residuals for the two variables flour‐like/cereal‐like and pea‐like/bean‐like are not homoscedastic. Therefore, a Brown‐Forsythe test was performed in addition, which yielded the same results as the ANOVA. Consequently, the results of the ANOVA, Kruskal‐Wallis test, and Brown‐Forsythe test are consistent for all attributes except sour: In the ANOVA and Brown‐Forsythe test, the differences in the sour attribute are significant (*p* = 0.037 and *p* = 0.039, respectively), while in the Kruskal‐Wallis test, the differences in the sour attribute are no longer significant (*p* = 0.083). However, small trends between the samples could be observed for *bitter*, *flour‐like/cereal‐like* and *rancid*. P3 was perceived as the most *sour*, but the least *bitter* and *flour‐like/cereal‐like* sample. P1 and P2 were rated as least *sour*, but as most *flour‐like/cereal‐like* and most *pea‐like/bean‐like*. In addition, P1 was rated as least *rancid*. P4 ranged somewhere between the other samples for *sour, bitter, flour‐like/cereal‐like and pea‐like/bean‐like*.

**FIGURE 2:**
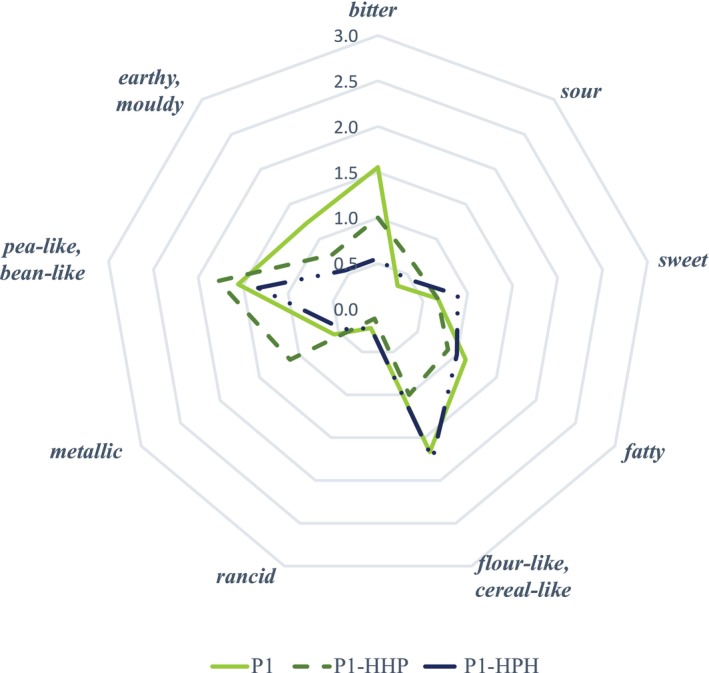
Comparison of intensity of aroma attributes for untreated P1 compared to pressure‐treated P1‐HPH and P1‐HHP.

To assess the impact of pressure treatment on the flavor profile of PPI, untreated P1 sample was compared to one HPH‐treated sample (P1‐HPH) and one HHP‐treated sample (P1‐HHP) in a descriptive sensory analysis using the same attributes as above (cf. Figure 2). ANOVA (*α* = 0.05) revealed no significant differences in the flavor attributes between the untreated, HPH‐treated, and HHP‐treated samples. The normal distribution of the residuals was examined using the Shapiro–Wilk test: For all 9 variables, the residuals do not follow a normal distribution. Therefore, a non‐parametric Kruskal‐Wallis test (*α* = 0.05) was performed in addition, which found no significant differences between the samples for all attributes. The homogeneity of variance/homoscedasticity was examined using the Levene test: The residuals for all 9 variables are homoscedastic. Consequently, the results of the ANOVA and Kruskal‐Wallis tests are consistent for all attributes. However, small trends between the samples could be observed: Both HPH‐ and HHP‐treated PPI samples (P1‐HPH and P1‐HHP) showed lower average intensities than the untreated P1 for the attributes *bitter* and *earthy/moldy*. Apart from this, P1‐HHP was perceived as more *metallic*, but less *flour‐like/cereal‐like* than untreated P1.

### 3.2 GC‐O and AEDA

The aim was to ensure comparability of the results for untreated and pressure‐treated PPIs as far as possible. Commercial pea protein isolate is available as dry powder. Further processing and use of PPI, including pressure treatment, involves the addition of water. For this reason, sample preparation and extraction of aroma‐active substances from the dry, powdered PPI was also carried out with the addition of water. A drying step after pressure treatment was explicitly omitted in order to rule out the influence of the additional drying process. To ensure consistent properties of the pressure‐treated aqueous suspensions, they were stored at approx. −20°C until sample preparation or sensory testing. In order to obtain the same weight‐ins for both the commercial PPI and the pressure‐treated samples produced from it, relative to the pure PPI, the required amount of suspension from PPI in water was calculated from the concentration of the suspension after HPH or HHP.

Aroma extract dilution analysis (AEDA) is an analytical method to determine the impact of an aroma‐active region and the corresponding aroma compound on the overall aroma profile of a sample. AEDA involves multiple GC‐O analyses of a dilution series of the original sample extract until no aroma‐active region is perceivable any more. These analyses result in the FD factors that indicate the highest dilution step at which a certain aroma attribute could be perceived. A higher FD means a presumably higher contribution of the aroma‐active region to the overall aroma (Grosch 1993).

In this work, dilutions ranging from $\frac{1}{1}$ (undiluted extract, FD factor 1) up to $\frac{1}{2048}$ (FD factor > 2048) of the original sample extract were investigated. At FD factor > 2048, only a few aroma‐active regions were still perceivable. FD factor < 1 means that the aroma‐active region was not perceived in the respective sample distillate. In this work, only FD factors ≥ 4 were taken into account and only the highest FD factors 512, 1024 and > 2048 were considered particularly relevant for the overall aroma.

GC‐O and AEDA revealed 144 aroma‐active regions with a FD factor ≥ 4 in the distillates of samples P3, P4, P1 and P2. The total number of observed aroma active regions differed between the different PPIs: 86 aroma‐active regions were observed for P2, only 74 regions for P1, 81 and 82 regions for P3 and P4, respectively. Among these were 17 regions with an FD factor of ≥ 512. Main attributes observed for aroma‐active regions were: *fatty, fruity, caramel‐like, flowery, green* and *pea‐like*.

Regarding only the areas with FDs ≥ 512, that are expected to have a major impact on the overall aroma of the sample, nine aroma‐active regions were observed for P2, only six for P1, ten regions for P3, and only five for P4. In comparison, in each of the pressure‐processed samples P1‐HPH and P1‐HHP, 12 aroma‐active regions with FD ≥ 512 were observed, twice as many as in the untreated P1 pea protein isolate.

For all investigated samples, a large number of aroma‐active regions could be observed via GC‐O and AEDA, with a broad variety of different aroma attributes. Regarding the untreated PPIs, P2 and P3 show the highest number of aroma‐active regions, especially with high FDs ≥ 512. This suggests that P2 and P3 are the untreated PPIs with the strongest aroma perception, which is not clearly visible from looking at the sensory results in Figure 1. Yet, similarly to the sensory evaluation in the panel, the AEDA rather shows small trends than major differences between the samples.

Considering the distillates of the four untreated PPI samples and, in addition, the distillates of the pressure‐treated samples P1‐HPH and P1‐HHP, a total number of 148 aroma‐active regions with a FD factor ≥ 4 was observed.

### 3.3 Identification of Aroma Compounds via GC–MS/O

36 aroma compounds were identified in the distillate of the samples, mainly carbonyls, carboxylic acids, pyrazines, esters, lactones, and other O‐heterocyclic compounds (cf. Table 2). Among these were 16 compounds with FD factors of FD ≥ 512 that are expected to have a major impact on the overall aroma of the samples: the unsaturated aldehydes (*E*)‐oct‐2‐enal (*fatty, soapy, grassy*), (*E,E*)‐2,4‐heptadienal (*fatty, fruity*), (*E,Z*)‐2,6‐nonadienal (*cucumber‐like*), (*E,Z*)‐2,4‐nonadienal (*fatty*) mostly contributed to the *fatty* aroma of the PPI samples. The pyrazines 2,3‐diethyl‐5‐methylpyrazine (*earthy, moldy*) and 3‐isobutyl‐2‐methoxypyrazine (*bell pepper‐like, pea‐like*) most likely contributed to the *earthy/moldy* and *pea‐like/bean‐like* aroma, respectively. The esters methyl 3‐methylbutanoate (*fruity, glue‐like*) and ethyl 3‐methylbutanoate (blueberry‐like, *fruity*) were likely responsible for the *fruity* aroma. The other identified compounds were hexanal (grassy), 3‐methylbutyl acetate (fruity, banana‐like), eugenol (clove‐like), the carboxylic acids 3‐methylbutanoic acid (*cheesy*) and nonanoic acid (*soapy, fatty, musty*), and some O‐heterocyclic compounds with a variety of different aromas (2‐pentylfuran (*mushroom‐like, fruity*), 3‐hydroxy‐2‐methylpyran‐4‐one (maltol; *caramel‐like*), 3‐hydroxy‐4,5‐dimethyl‐5*H*‐furan‐2‐one (sotolone; *savory, celery‐like*)). Substances described in previous literature in PPIs or peas are in bold letters (Bi et al. 2020; Ebert et al. 2022; El Youssef et al. 2020; Fischer et al. 2022; Murat et al. 2013; Murray et al. 1976; Murray et al. 1968; Trikusuma et al. 2020; Trindler et al. 2022; Zhogoleva et al. 2023). Sotolone was previously reported in lupines, another type of legumes, with high FD factors (Bader et al. 2009).

**TABLE 2:** Aroma‐active regions and aroma compounds identified in the PPI samples (only regions observed at FD ≥ 128 in at least one sample were considered in the table; gray: Aroma‐active regions with the highest contributions to the overall aroma).

Nr^a^	RI^b^		Odorant	CAS number	Odor quality^c^	FD^d^						IP^e^
	DB‐FFAP	DB‐5				P3	P4	P2	P1	P1‐HPH	P1‐HHP	
1	< 1000		*Unknown*		Malty, nutty, solvent‐like	128	8	1	1	4	64	
2	< 1000		Butane‐2,3‐dione	431–03‐8	Butter‐like	128	32	8	16	16	8	RI, O
3	1042	806	Methyl 3‐methylbutanoate	556–24‐1	Fruity, glue‐like	512	< 1	2	< 1	32	2	RI, O
4	1075	852	Ethyl 3‐methylbutanoate	108–64‐5	Blueberry‐like, fruity	256	128	1024	128	32	512	RI, O, MS
5	1082	800	hexanal	66–25‐1	Grassy	4	2	8	1	512	≥ 2048	RI, O, MS
6	1115	882	3‐methylbutyl acetate (isoamyl acetate)	123–92‐2	Fruity, banana‐like	< 1	2	8	< 1	2	≥ 2048	RI, O, MS
7	1153		*Unknown*		Burnt, gas‐like, sulfurous	128	8	< 1	< 1	< 1	< 1	
8	1180	888	Heptan‐2‐one	110–43‐0	Blue mold cheese‐like, glue‐like, fruity	4	< 1	128	32	64	< 1	RI, O, MS
9	1219	995	2‐pentylfuran	3777‐69‐3	Mushroom‐like, fruity	128	32	512	32	64	16	RI, O, MS
10	1227		*Unknown*		Green, fatty, geranium‐like	128	16	8	32	< 1	< 1	
11	1288	984	Oct‐1‐en‐3‐one	4312‐99‐6	Mushroom‐like	256	32	32	32	32	64	RI, O
12	1314	926	(2,5 or 2,6)‐dimethylpyrazine	123–32‐0/108–50‐9	Bread‐like, nutty, malty, roasty	256	256	2	32	16	16	RI, O, MS
13	1333		2‐acetyl‐1‐pyrroline (1‐(3,4‐dihydro‐2*H*‐pyrrol‐5‐yl)ethanone)	85,213–22‐5	Popcorn‐like, roasty	4	< 1	128	< 1	2	< 1	RI, O
14	1388	1005	*Unknown*		Fatty, nutty, earthy	64	128	8	4	32	128	RI, O, MS
15	1413	1047	*Unknown*		Nutty, rancid	< 1	256	< 1	< 1	< 1	< 1	RI, O
16	1425	1053	(*E*)‐oct‐2‐enal	2548‐87‐0	Fatty, soapy, grassy	512	< 1	≥ 2048	512	≥ 2048	512	RI, O, MS
17	1444	911	Methional (3‐methylsulfanylpropanal)	3268‐49‐3	Cooked potato‐like	128	16	64	32	< 1	64	RI, O
18	1473	1150	2,3‐diethyl‐5‐methylpyrazine	18,138–04‐0	Earthy, moldy	512	2	64	8	128	128	RI, O
19	1481		*Unknown*		Green, fatty, soapy	256	8	< 1	256	512	128	RI, O
20	1488	1161	3‐*s*‐butyl‐2‐methoxypyrazine (2‐butan‐2‐yl‐3‐methoxypyrazine)	24,168–70‐5	Pea‐like	64	256	< 1	< 1	< 1	< 1	RI, O
21	1497		(*E,E*)‐2,4‐heptadienal	4313‐03‐5	Fatty, fruity	256	64	≥ 2048	64	≥ 2048	1024	RI, O, MS
22	1500	1183	3‐isobutyl‐2‐methoxypyrazine (2‐methoxy‐3‐(2‐methylpropyl)pyrazine)	24,683–00‐9	Bell pepper‐like, pea‐like	< 1	256	128	1024	≥ 2048	512	RI, O
23	1514	1294	*Unknown*		Green, green bell pepper‐like, fruity	256	< 1	8	< 1	≥ 2048	128	
24	1527	1160	(*E*)‐non‐2‐enal	18,829–56‐6	Fatty, cardboard‐like	2	128	8	256	16	32	RI, O
25	1530		*Unknown*		Popcorn‐like, caramel‐like	< 1	128	< 1	< 1	< 1	< 1	
26	1553	1265	*Unknown*		Red currant‐like	128	< 1	< 1	< 1	< 1	< 1	
27	1553		*Unknown*		Fatty, pea‐like	8	32	256	< 1	64	32	
28	1564	1150	(*E,Z*)‐2,6‐nonadienal	557–48‐2	Cucumber‐like	4	4	≥ 2048	512	128	512	RI, O
29	1608	818	Butanoic acid	107–92‐6	Vomit‐like, cheesy	256	32	< 1	< 1	< 1	< 1	RI, O, MS
30	1621	1068	3‐methylbenzaldehyde	620–23‐5	Honey‐like, caramel‐like, flowery	256	8	32	1	2	< 1	RI, O, MS
31	1629	1253	(*E*)‐dec‐2‐enal	3913‐81‐3	Coriander‐like, fatty, waxy	128	< 1	< 1	8	16	< 1	RI, O, MS
32	1636	900	*UNKNOWN*		Flowery	< 1	< 1	128	< 1	< 1	< 1	
33	1643	1053	acetophenone (1‐phenylethanone)	98–86‐2	Marzipan‐like, flowery	< 1	< 1	256	8	< 1	1	RI, O, MS
34	1650	1200	(*E,Z*)‐2,4‐nonadienal	21,661–99‐4	Fatty	1024	1024	≥ 2048	≥ 2048	≥ 2048	≥ 2048	RI, O
35	1664	871	3‐methylbutanoic acid	503–74‐2	Cheesy	512	1024	64	32	16	64	RI, O, MS
36	1693	1074	*Unknown*		Fruity, caramel‐like, flowery	512	512	1024	128	1024	64	
37	1708		*Unknown*		Citrus‐like, red currant‐like	128	256	128	64	512	256	
38	1754		Unknown		Bitter almond‐like	< 1	< 1	< 1	< 1	1024	512	
39	1777		*Unknown*		Fruity, caramel‐like, flowery	≥ 2048	< 1	512	< 1	< 1	< 1	
40	1800		*Unknown*		Caramel‐like, flowery, fruity	< 1	≥ 2048	8	256	512	128	
41	1823		*unknown*		Fatty	< 1	< 1	< 1	256	< 1	< 1	
42	1833	1265	Geraniol ((*E,E*)‐3,7‐dimethylocta‐2,6‐dien‐1‐ol)	106–24‐1	Flowery	128	< 1	< 1	< 1	< 1	< 1	RI, O, MS
43	1838	1427	α‐ionone ((*E*)‐4‐(2,6,6‐trimethyl‐1‐cyclohex‐2‐enyl)but‐3‐en‐2‐one)	127–41‐3	Fruity, raspberry‐like	128	1	< 1	128	1	1	RI, O
44	1850	1100	guaiacol (2‐methoxyphenol)	90–05‐1	Smoky, smoked ham‐like	256	128	64	64	128	16	RI, O, MS
45	1867		*Unknown*		Flowery, fruity, soapy	256	32	< 1	< 1	< 1	< 1	
46	1908	1265	γ‐octalactone (5‐butyloxolan‐2‐one)	104–50‐7	Coconut‐like	128	64	128	32	16	128	RI, O, MS
47	1954	1106	maltol (3‐hydroxy‐2‐methylpyran‐4‐one)	118–71‐8	Caramel‐like	512	128	64	64	64	64	RI, O, MS
48	1985		*Unknown*		Flowery, honey‐like, rose‐like	8	2	128	16	1	< 1	
49	2000		*Unknown*		Flowery, musty, green	512	256	128	128	256	128	
50	2009	1074	furaneol (4‐hydroxy‐2,5‐dimethyl‐2*H*‐furan‐3‐one)	3658‐77‐3	Caramel‐like, strawberry‐like	128	32	256	< 1	< 1	2	RI, O
51	2018	1360	γ‐nonalactone (5‐pentyloxolan‐2‐one)	104–61‐0	Coconut‐like	64	256	8	32	64	128	RI, O, MS
52	2082	1081	*p*‐cresol (4‐methylphenol)	106–44‐5	Horse stable‐like, fecal	64	16	128	32	4	16	RI, O, MS
53	2100	1089	*Unknown*		Leather‐like, phenolic, smoky	128	32	< 1	1	< 1	4	RI, O, MS
54	2145		Nonanoic acid	112–05‐0	Soapy, fatty, musty	< 1	< 1	< 1	< 1	< 1	512	RI, O, MS
55	2155	1172	Eugenol (2‐methoxy‐4‐prop‐2‐enylphenol)	97–53‐0	Clove‐like	128	64	16	512	256	≥ 2048	
56	2185	1106	Sotolone (3‐hydroxy‐4,5‐dimethyl‐5*H*‐furan‐2‐one)	28,664–35‐9	Savory, celery‐like	≥ 2048	≥ 2048	512	1024	≥ 2048	≥ 2048	RI, O
57	2264		*Unknown*		Herbal, red currant‐like	128	< 1	< 1	< 1	< 1	< 1	
58	2467	1400	Skatol (3‐methyl‐1*H*‐indole)	83–34‐1	Fecal, mothball‐like	128	64	64	16	8	64	RI, O, MS

*Note:* Shaded values highlight aroma‐active regions with the highest contributions to the overall aroma impression.

^a^ Consecutive numbering of odorants according to their retention indices on capillary column DB‐FFAP.

^b^ Linear retention index on the DB‐FFAP or DB‐5 capillary column.

^c^ Odor quality perceived at the odor detection port by three trained panelists.

^d^ Flavor dilution factor determined on DB‐FFAP. FD factor < 1 means that the aroma‐active region was not perceived in the respective sample distillate.

^e^ Identification procedure: RI: Odorant was identified by comparison of its retention indices on capillaries DB‐FFAP and/or DB‐5 with data of pure reference compounds. O: Odorant was identified by comparison of its odor quality with data of pure reference compounds. MS: Odorant was identified by comparison of its mass spectrum (EI mode) with data of pure reference compounds.

For several aroma compounds (hexanal, (*E,E*)‐2,4‐heptadienal, sotolone, and several unknown compounds (compounds #23, #37, and #38)), higher FD factors were observed for both HPH‐ and HHP‐treated samples compared to the untreated PPI: The FD of hexanal strongly increased from FD 1 in untreated P1 to FD 512 in P1‐HPH and FD ≥ 2048 in P1‐HHP (cf. Table 2). For (*E,E*)‐2,4‐heptadienal, an increase from FD 64 in the untreated PPI to FD ≥ 2048 in the HPH‐treated sample and FD 1024 in the HHP‐treated sample was observed. Compound #23 (unknown; *green, green bell‐pepper‐like, fruity*) was not observed in untreated P1 (FD < 1), but was present in P1‐HPH (FD ≥ 2048) and in P1‐HHP (FD 128). Similarly, compound #38 (unknown; *bitter almond‐like*) was not perceivable in untreated P1, but in P1‐HPH (FD 1024) and in P1‐HHP (FD 512). For compound #37 (unknown; *citrus‐like, red currant‐like*), an increase from FD 64 in the untreated P1 to FD 512 in the HPH‐treated sample and FD 256 in the HHP‐treated sample was observed. For sotolone, the FDs increased from FD 1024 in the untreated PPI to FD ≥ 2048 (HPH (60/3)) and FD ≥ 2048 (P1‐HHP). For other aroma compounds ((*E*)‐oct‐2‐enal, 3‐isobutyl‐2‐methoxypyrazine, compound #19 (unknown; *green, fatty, soapy*), compound #36 (unknown; *fruity, caramel‐like, flowery*), and #40 (unknown; *caramel‐like, flowery, fruity*)), an increase of the FD was only observed for HPH processing, whereas the FD remained unchanged or slightly decreased in the HHP‐treated pea protein isolate. The aldehyde (*E,Z*)‐2,4‐nonadienal was present in untreated, HPH‐treated, and HHP‐treated samples in equally high FD ≥ 2048. The compounds 3‐methylbutyl acetate and nonanoic acid were not perceived in the untreated or HPH‐treated sample, but appeared in the HHP‐treated sample with high flavor dilution factors of FD ≥ 2048 and FD 512, respectively. For some compounds (ethyl 3‐methylbutanoate, (*E,Z*)‐2,6‐nonadienal), a decrease of the FD was observed for HPH‐treated samples, while no change or even an increase of the FD was perceived for the HHP‐treated sample. For (*E*)‐non‐2‐enal and compound #41 (unknown; *fatty*), the FD was lower in both the HPH‐ and HHP‐treated sample.

## 4 Discussion

Regarding the four non‐pressure treated PPI samples, the flavor attributes perceived with highest intensities were the aroma attributes *pea‐like/bean‐like, flour‐like/cereal‐like*, and *fatty* and the taste attributes *bitter* and *sour*. Some compounds were detected at high FDs in all four PPIs, suggesting that these compounds play an important role in their overall aroma: the unsaturated aldehyde, (*E,Z*)‐2,4‐nonadienal (*fatty*), and the O‐heterocyclic compound sotolone (*savory, celery‐like*). Some other unsaturated aldehydes, while not perceived in all samples at equally high FDs, are expected to have a high impact on the *fatty and green* aroma: (*E*)‐oct‐2‐enal, (*E,E*)‐2,4‐heptadienal, and (*E,Z*)‐2,6‐nonadienal. The compounds with *fatty* or *pea‐like/bean‐like* aroma identified by GC–MS/O are expected to directly contribute to the corresponding attributes *fatty* or *pea‐like/bean‐like* in the sensory evaluation, respectively. For *flour‐like/cereal‐like*, the most intense attribute within the retronasal aroma and taste profile, no distinct compounds could be identified that directly contribute to this aroma impression. In the case of white and whole wheat flour, the characteristic aroma is known to be caused by a combination of multiple aroma‐active compounds rather than by single aroma‐active compounds (Czerny and Schieberle 2002). Multiple relevant compounds reported in white and whole wheat flour (Czerny and Schieberle 2002) were also found in high FDs in this work: sotolone, (*E*)‐non‐2‐enal, (*E,Z*)‐2,6‐nonadienal, and 3‐methylbutanoic acid. It is possible that in a similar way, a combination of those compounds is responsible for the *flour‐like/cereal‐like* aroma attribute perceived in the PPIs. Furthermore, some aroma‐active regions with *bread‐like* aroma were detected in FDs < 128, possibly also contributing to the *flour‐like/cereal‐like* aroma.

No significant differences were found between the four PPIs, neither in relation to the FDs of the aroma‐active compounds nor in the perceived intensities of the aroma attributes. A significant difference was found only for the *sour* taste attribute. The results of the instrumental analysis are consistent with the results of the sensory analysis. Aroma attributes are triggered by aroma‐active, volatile compounds. Taste attributes, on the other hand, are elicited by non‐volatile substances. Gas chromatographic methods can only analyze volatile substances. It is therefore conceivable that some substances underlying the *sour* taste attribute remained undetected using GC–MS. In accordance with the similar aroma profiles in the sensory analysis, analysis via GC‐O, AEDA and GC–MS/O revealed that the four different untreated PPIs investigated in this work contain mostly the same aroma‐active compounds. Only the contribution of single aroma compounds to the overall aroma varied between the four different PPI samples to some extent. The small differences in the aroma profiles seen in the sensory evaluation (cf. 3.1) could be caused by different cultivars (Azarnia et al. 2011; Cui et al. 2020; Lam et al. 2018), different extraction methods (Cui et al. 2020) and parameters or different drying methods and parameters (Sumner et al. 1981). The cultivar of the pea, for example, determines the composition of aroma‐active substances and precursors (e.g., fats/fatty acids) in the pea raw material (Trindler et al. 2022). The extraction method and parameters used (e.g., pH, salt concentration, temperature) influence which aroma‐active compounds and precursors from the pea raw material are dissolved in the processing water and removed, and which compounds remain in the pea protein isolate. One reason is that chemical and enzymatic treatments can change the protein structure, which affects the affinity of the protein towards the different aroma compounds (Wang and Arntfield 2016). Another possible reason might be that pH, salt concentration and temperature can modify the solubility of a certain aroma compound. For example, a higher pH could facilitate transfer of *sour* carboxylic acids from the protein raw material into the processing water. This could explain the low intensity of *sour* in PPIs P1 and P2. After the extraction, the drying method, specifically the temperature and duration of heat exposure, can impact the composition of aroma‐active substances and precursors in pea protein isolates. This relationship is more complex than it seems: On the one hand, spray‐drying of pea protein isolates was reported to be more suitable for the reduction of off‐flavors compared to freeze‐drying and drum‐drying (Sumner et al. 1981). On the other hand, it was observed that while spray‐drying reduces the overall volatile organic compounds (VOC) concentration in PPIs, it increases the concentration of aldehydes (Ma et al. 2016). Aldehydes, however, contribute largely to the typical *beany, pea‐like* aroma of PPI. Unsaturated carbonyls are well‐known oxidation products of unsaturated fatty acids and derive from linoleic acid and linolenic acid (Murray et al. 1976). Furthermore, it is known that heat induces reactions such as Maillard‐reaction (formation of e.g., 2‐acetyl‐1‐pyrroline) or Strecker‐degradation (formation of e.g., methional and 3‐methylbutanal) (Tairu et al. 2000), leading to typical roasty and malty aroma compounds. Methional and 3‐methylbutanal were reported as key odorants in barley malt (Fickert and Schieberle 1998). As a result, the higher intensity of *flour‐like/cereal‐like* in P1 and P2 could originate from exposition to a higher temperatures or heating for a longer period of time than the other PPIs.

For both HPH and HPP, there is a wide range of possible parameters. The key parameters for HPH are the pressure and the number of cycles, that is, passes through the system. HPH is usually carried out at pressures between 50 and 200 MPa; below this, it is simple homogenization, above this, it is referred to as ultra‐high pressure homogenization (UHPH) (Levy et al. 2021; Sherman et al. 2024). With the equipment used in this study, an APV Lab Homogenizer 2000 (SPX FLOW, Charlotte, USA), pressures of up to 2000 bar/200 MPa are possible. With HPH, the temperature of the process material also increases with increasing pressure. Since this study was intended to investigate the influence of non‐thermal processes on aroma, a comparatively low pressure of 60 MPa was intentionally used for HPH in order to minimize the heating of the process material and possible influences of temperature on the aroma. In the case of HHP, the key parameters are the pressure applied and the duration of exposure in the chamber, usually a few minutes between 100 and 1000 MPa (Aganovic et al. 2021). The Hiperbaric L135 chamber (Hiperbaric, Burgos, Spain) used in this study can reach pressures of up to 6000 bar/600 MPa. In this work, the maximum pressure of 600 MPa was used, as it was assumed that, if any, a change would occur at this pressure. Due to the large volume of the water tank, an increase in temperature during pressure treatment using HHP does not play as much of a role as with HPH, so that the maximum pressure of 600 MPa could be used here.

Various parameters were tested. For the sensory and analytical evaluation, however, the samples with the maximum pressure and the maximum number of cycles or the maximum exposure time were first examined from the tests carried out, as it was assumed that, if any, a change would occur in these samples. Since no change could be observed even under these conditions, the PPIs treated under milder conditions were not examined in more detail. In addition, one of the commercial PPIs (P4) did not dissolve in water at all; instead, the protein immediately settled, meaning that there was no stable suspension that could be pumped through the homogenizer. Sample P4 could therefore not be treated with HPH. HHP treatment would be possible, but the data for P4 would still be incomplete.

Comparing the flavor profiles of untreated and pressure‐treated PPI samples, only some minor, non‐significant changes were observed: Both the P1‐HPH and P1‐HHP were described as slightly less *bitter* and *earthy/moldy* than untreated P1 sample. HHP‐treated sample P1‐HHP was perceived as more *metallic* and slightly less *flour‐like/cereal‐like* than untreated P1. On the contrary, a strong increase in the FDs was observed for (*E,E*)‐2,4‐heptadienal, hexanal, and nonanoic acid for both pressure treatments.

High‐pressure treatment of proteins can cause changes in protein structure. When considering the influence of pressure on the flavor compound–protein interaction, it is important to take into account the type of interactions involved. Pressure treatment usually has no effect on covalent bonds between proteins and flavor compounds (Vatansever et al. 2024). However, pressure treatment may influence non‐covalent interactions, which in turn could affect the release of flavor compounds from the protein matrix and the FDs in which the flavor compounds are perceived. Weakening the flavor compound–protein interaction should lead to greater extraction of this flavor compound and thus to a higher FD; conversely, strengthening the flavor compound–protein interaction should reduce the FD. It would also be expected that weaker interactions would be more easily overcome than stronger ones.

For many substances ((*E,Z*)‐2,4‐nonadienal, 2,5−/2,6‐dimethylpyrazine, 3‐*s*‐butyl‐2‐methoxypyrazine, 3‐isobutyl‐2‐methoxypyrazine, 2‐pentylfuran, maltol), no significant change in FD was observed as a result of pressure treatment; the interaction between the protein and these substances became neither stronger nor weaker. For some substances, that is, ((*E,E*)‐2,4‐heptadienal, hexanal, nonanoic acid), the observed FD after pressure treatment (both HPH and HHP) was distinctively higher, suggesting that the interaction between protein and aroma compound was reduced. Overcoming the interaction would be expected in particular for weaker interactions, such as between alcohols or esters and the protein. Instead, however, this case was observed with the aldehydes (*E,E*)‐2,4‐heptadienal and hexanal, which exhibit relatively strong hydrophobic interactions with the protein.

For (*E*)‐2‐octenal, a distinct increase in FD was observed after HPH treatment only, while for 3‐methylbutyl acetate, an increase in FD was observed for HHP treatment only. It is unclear why one of the pressure treatments caused a marked increase in FD, that is, a presumed distinct reduction in the aroma substance‐protein interaction, while the other pressure treatment caused no change. For the lactone sotolone, which can interact with the protein primarily via hydrogen bonds, the FD was slightly higher after HPH or HHP treatment, suggesting a slight weakening of the interactions. The opposite effect was observed for three substances: after pressure treatment with HPH, for ethyl 3‐methylbutanoate, (*E,Z*)‐2,6‐nonadienal, (*E*)‐2‐nonenal, or HHP‐treatment, for (*E*)‐2‐nonenal, the FD was slightly lower than before. In this case, pressure treatment appears to have increased the interaction. Surprisingly, in addition to the ester ethyl 3‐methylbutanoate, this was also the case with (*E,Z*)‐2,6‐nonadienal and (*E*)‐2‐nonenal, which, as aldehydes with longer carbon backbone, can readily form strong hydrophobic interactions with the protein anyway. Greater retention of flavor compounds by the protein matrix could reduce off‐flavors. Instead, pressure treatment released some compounds rather than retaining them, but this did not have a significant effect on the flavor profile. Flavor compounds released by the pressure treatment might, however, be removed by a subsequent washing procedure.

The differences in the results between analytical and sensory evaluation could also be partly due to the methods used: AEDA is highly dependent on the extraction protocol, mainly sample amount, solvent volume, and volume after destillation. AEDA is an untargeted analysis method that can provide an estimation of the differences in the FDs between different samples but does not reveal quantitative data about the concentration of analytes in the sample. This requires additional quantification, for example, via stable isotope dilution assay (SIDA). SIDA allows examining if the changes in some FD factors are confirmed by changes in the concentration of the corresponding analytes. As no significant flavor changes were observed in the sensory evaluation after pressure treatment, no quantification of aroma compounds via SIDA was included in this work. In sensory evaluation, samples can only be presented in pure form or in food matrices, whereby pure water offers the advantages of being odorless and tasteless, resembles the saliva already present in the mouth, and does not alter the texture and mouthfeel due to its low viscosity. In addition, samples in sensory analysis are presented either pure, that is, undiluted, or diluted in the matrix (water). In this case, a relatively diluted suspension of pea protein (0.6% (w/w)) in water was used, as preliminary tests had shown that this concentration was best for revealing differences in the flavor profile. The panelists then tasted only a few sips of this diluted suspension. In contrast, AEDA uses a highly concentrated extract in a completely different solvent (DCM) in order to be able to analytically detect as many flavor compounds as possible, including those present in very low concentrations in the sample, since flavor compounds in low concentrations but with low odor thresholds can be relevant to the aroma of a sample. In this study, 50.00 g of PPI was extracted in 50 mL of water with 150 mL of DCM, and the volume of the solvent extract was reduced to 100 μL, of which 2 μL was injected undiluted for the FD1 measurement. However, this also means that odor impressions of aroma substances may be found in the AEDA that are below their odor threshold in the native sample and therefore are not relevant to the sensory profile of the sample. Differences in the FDs of substances between different samples may appear more dramatic and important in the AEDA than they really are for the aroma profile of the sample. To test this hypothesis in a future work, solvent extraction, SAFE and distillation could be done using a suspension of PPI in water in the same concentration as in the sensory evaluation, or sensory evaluation with a higher sample concentration could be applied. However, each of these variations will come with their own intricacies and limitations. In addition, less polar analytes such as hexanal (Log *p* = 1.65) or (*E,E*)‐2,4‐heptadienal (Log *p* = 1.73) (Saffarionpour 2024) dissolve poorly in water and could therefore remain in the PPI matrix during sensory testing, whereas DCM is used during sample preparation for analysis by solvent extraction, which can also dissolve the nonpolar analytes from the PPI matrix.

A small increase in the FDs upon pressure treatment or an increase for one of the treatments only was observed for sotolone, (*E*)‐oct‐2‐enal and 3‐methylbutyl acetate. No or almost no change in FDs was observed for (*E,Z*)‐2,4‐nonadienal, 2,5−/2,6‐dimethylpyrazine, 3‐*s*‐butyl‐2‐methoxypyrazine, 3‐isobutyl‐2‐methoxypyrazine and some *unknown* compounds. For (*E,Z*)‐2,6‐nonadienal, a slight decrease of FD was noticed for HPH treatment. The aroma profile of the pressure‐treated PPI was described as slightly less fatty, but no significant (*α* = 0.05) difference was found. The FDs of the identified substances with a fatty aroma also show no clear change: While the FD of (*E*)‐non‐2‐enal was lower after the pressure treatments, the FD of (*E,Z*)‐2,4‐nonadienal remained the same and the FDs of (*E,E*)‐2,4‐heptadienal and nonanoic acid were even higher in the pressure‐treated samples than in the untreated sample. The present investigations show that neither HPH nor HHP significantly changed the overall flavor profile or sensory character of the PPI investigated in this study, meaning that no negative changes were observed (under these parameters, with this protein). However, it should be noted that only one set of parameters for HPH and HHP has been examined at one PPI so far. This raises the question of whether the previous observation also applies to variations in PPI, pressure, and number of cycles in HPH or treatment duration in HHP. Based on the findings of this study, these pressure treatments are therefore likely to be suitable if the techno‐functional properties (solubility, water absorption, gelling properties, foaming capacity, oil holding capacity, etc.) of the proteins are to be altered by pressure treatment without impairing the flavor, or if pressure treatment is to be used for the production of a foodstuff or for preservation purposes.

## 5 Conclusion and Outlook

Four commercial PPIs were compared regarding their flavor profile and aroma compounds using sensory evaluation and instrumental analysis. The samples were described as mainly *pea‐like/bean‐like*, *flour‐like/cereal‐like*, *bitter*, *fatty* and *sour* and were rather similar in their flavor profiles, with mainly non‐significant differences between the samples. Unsaturated aldehydes, typical products of lipid oxidation, were identified as major contributors to the overall *fatty, green* aroma. (*E,Z*)‐2,4‐Nonadienal (*fatty*) and sotolone (*savory, celery‐like*) were detected with high FDs in all of the four different PPIs, suggesting that these compounds play an important role in the overall aroma. In addition, (*E*)‐oct‐2‐enal, (*E,E*)‐2,4‐heptadienal and (*E,Z*)‐2,6‐nonadienal are also expected to have a high impact on the *fatty* aroma of the PPI samples.

The influence of pressure‐treatment by high‐pressure homogenization or high hydrostatic pressure on the flavor profile and aroma compounds of PPI was investigated. Neither of the pressure treatments significantly changed the overall flavor profile and sensory character of the pea protein isolate. No negative changes of the flavor were observed for the parameters considered here. This study contributes to understanding of aroma of pea protein isolates after high‐pressure treatment. In the future, the method used here could be supplemented by a quantitative determination of selected analytes and the calculation of their odor activity values (OAV) from the measured concentrations and their odor thresholds (from the literature). It would also be interesting to see whether the observations made in this study can be confirmed for other PPIs and other HPH or HHP parameters to further evaluate whether pressure treatment of PPIs using HPH or HHP is generally suitable for altering the techno‐functional properties of pea proteins, production of a foodstuff or for preservation purposes, without affecting the flavor profile.

## Author Contributions

**Gina Zeh:** writing – review and editing, supervision. **Christina Hopf:** writing – review and editing, writing – original draft, investigation, visualization, data curation, formal analysis. **Laura Scheuer:** writing – review and editing, resources, investigation. **Lea Friedrich:** conceptualization, writing – review and editing, writing – original draft, investigation, visualization, data curation, formal analysis. **Pauline Fauquet:** writing – review and editing, resources, investigation.

## Funding

This research was conducted within the framework of the 31st CORNET Call under the research project “ProTInnov—Improving structure and flavor performance for healthier plant‐based food using innovative approaches”. The authors gratefully acknowledge the funding of the Service Public de Wallonie—Economie, Emploi, Recherche (SPW EER) and the AiF within the program for promoting the Industrial Collective Research (IGF) of the Federal Ministry of Economic Affairs and Climate Action (BMWK), based on a resolution of the German Parliament. Project AiF 321 EN.

## Consent

Informed consent was obtained from all subjects participating in the sensory study.

## Conflicts of Interest

The authors declare no conflicts of interest.

## Data Availability

The data that support the findings of this study are available from the corresponding author upon reasonable request.
